# Soft Tissue Masses of Hand: A Radio-Pathological Correlation

**DOI:** 10.1155/2015/752054

**Published:** 2015-09-02

**Authors:** Aditi Agarwal, Mahesh Prakash, Pankaj Gupta, Satyaswarup Tripathy, Nandita Kakkar, Radhika Srinivasan, Niranjan Khandelwal

**Affiliations:** ^1^Department of Radiodiagnosis and Imaging, Postgraduate Institute of Medical Education and Research (PGIMER), Chandigarh 160012, India; ^2^Department of Plastic Surgery, Postgraduate Institute of Medical Education and Research (PGIMER), Chandigarh 160012, India; ^3^Department of Histopathology, Postgraduate Institute of Medical Education and Research (PGIMER), Chandigarh 160012, India; ^4^Department of Cytopathology, Postgraduate Institute of Medical Education and Research (PGIMER), Chandigarh 160012, India

## Abstract

*Aim*. To evaluate soft tissue masses of the hand with magnetic resonance imaging (MRI) and ultrasonography (USG) and to correlate imaging findings with pathological findings. *Material and Methods*. Thirty-five patients with soft tissue masses of the hand were evaluated with high resolution USG and contrast enhanced MRI of the hand, prospectively over a period of 2.5 years. The radiological diagnosis was then compared with cytology/histopathology. *Results*. There were a total of 19 (55%) females. The mean age was 27.45 ± 14.7 years. Majority (45%) of cases were heteroechoic. Four cases were predominantly hyperechoic. These were later diagnosed as lipomas. Four cases were anechoic (diagnosed as ganglions). Only four lesions showed hyperintense signal on T1-weighted images. Out of these, 3 were lipomas and one was cavernous haemangioma. Three lesions were hypointense on T2-weighted images. All these lesions were diagnosed as giant cell tumor of the tendon sheath. A correct diagnosis was possible on MRI in 80% of cases (*n* = 28). *Conclusion*. MRI provides specific findings for diagnosis of certain soft tissue lesions of the hand. Ultrasonography allows accurate diagnosis of hemangioma/vascular malformations. However, in most conditions, imaging findings are nonspecific and diagnosis rests on pathologic evaluation.

## 1. Introduction

Common soft tissue masses of hand include ganglia, giant cell tumor of tendon sheath (GCTTS), lipomas, nerve sheath tumors, glomus tumors, and hemangiomas [1]. High-resolution ultrasonography (USG) and magnetic resonance imaging (MRI) play an important role in characterisation of these masses. Besides, they provide information about the site, size, extent, and relation with surrounding structures. Previous studies have shown the efficacy of imaging in the characterisation of soft tissue masses in this location [1–6]. However, these studies have been limited by the patient number, variable sensitivity and specificity, and lack of uniform protocols. Besides, several advancements in imaging techniques particularly MRI are expected to have an impact on the evaluation of soft tissue masses. We conducted a prospective study to evaluate soft tissue masses in the hand using high resolution USG and MRI. A correlation with pathological diagnosis was also conducted.

## 2. Material and Methods

This prospective study was conducted over a period of 2.5 years from 1 July 2011 to 31 December 2014. The study was approved by institute ethics committee and informed written consent was obtained from all patients. A total of 35 patients presenting with soft tissue masses of the hand to the plastic surgery outpatient department were included. All patients underwent high resolution USG and contrast enhanced MRI of the hand. Patients with contraindication for MRI and those with history of previous MRI contrast reaction or renal failure and inconclusive histopathology/cytology were excluded from study.

### 2.1. Imaging

MRI was performed on a 3-Tesla scanner (Magnetom Verio, Siemens Medical Systems) using a dedicated wrist coil. The patients were scanned in the most comfortable position (as uniform positioning in each patient was not feasible in view of the hand swelling). T1-weighted (TR/TE, 650–1070/10–20), axial, coronal, and sagittal, T2-weighted (TR/TE, 3600–4324/80–98) axial, coronal and/or sagittal and axial, coronal, and sagittal gadolinium enhanced (following intravenous injection of 0.1 mL/kg of gadopentetate dimeglumine, maximum 10 mL) T1-weighted (TR/TE, 650–950/10–20) sequences were performed. Images were reviewed by two radiologists with 10 years and 3 years of experience in musculoskeletal MRI in consensus. The same radiologist performed B-mode as well as color Doppler USG of the masses using Philips HD 11 XE and iU22 machines equipped with a 3-to-12 MHZ linear transducer Doppler settings that were optimized to “low flow,” with a medium wall filter (to minimize flash artifact) and a pulse repetition frequency of 700 Hz. The color gain was adjusted to just below the noise floor and maintained at this level throughout the scanning protocol. Each swelling was scanned in various planes. The correlation with pathological findings was performed only for MRI and ultrasonography was used as an adjunct imaging modality.

The radiologists were blinded to the clinical diagnosis/pathological findings. All patients underwent fine needle aspiration cytology (FNAC) and/or biopsy. The radiological diagnosis was then compared with cytology/histopathology.

The standard of reference was cytology/histopathology in all the patients. The efficacy of the radiological investigations was determined by comparing USG and MRI findings with the histopathological/cytological diagnosis.

### 2.2. Statistical Analysis

The statistical analysis was carried out using Statistical Package for Social Sciences (SPSS Inc., Chicago, IL, version 15.0 for Windows). All quantitative variables were estimated using measures of central location (mean and median) and measures of dispersion (standard deviation and standard error). Qualitative or categorical variables were described as frequencies and proportions (percentages).

## 3. Results

Out of the 35 patients in the study group, there were a total of 19 (55%) females and 16 (45%) male patients. The mean age of the sample population was 27.45 ± 14.7 years (range 3–61 years).

### 3.1. Clinical Features

All the patients (*n* = 35) presented with soft tissue swelling in the hand with mean size of 3.12 ± 2.1 cms (range 0.5–8 cms). Ten (28.5%) patients had associated pain. Four of the thirty patients (11.5%) also had restriction of finger movement. There was a wide range of difference in the duration of the swelling in the study group. The mean duration of swelling was 3.5 years (range 2 months to 20 years). Four (11.5%) patients had swelling since birth which was gradually increasing in size. In 21 (60%) patients swelling was located of the ventral surface of the hand. Five (15%) had swelling on the dorsal aspect; in the rest of the 9 (25%) patients it involved both dorsal as well as the ventral aspect. In 16 (45%) patients it was located in the fingers, 12 (35%) had swelling in the palm, 5 (15%) involved both palm and wrist, and in 2 (5%) it was located in the web space. In 50% of the patients the swelling was well-defined. Twenty-five (70%) swellings were soft in consistency and the rest 10 (30%) were firm in nature. Twenty-one (60%) swellings were noncompressible while 14 (40%) were compressible. In 5 (15%) cases, swellings were found in relation to the tendons of the hand. Three of them were GCTTS and two were due to the presence of chronic tenosynovitis.

### 3.2. Imaging Findings

The swellings included in this study demonstrated variable echogenicity on gray scale. The maximum number (45%) of cases was heteroechoic. Four cases included in the study were predominantly hyperechoic in echogenicity. These were later diagnosed as lipomas. Four cases were anechoic in appearance. These cases were diagnosed as ganglions. There was presence of increased color flow in 7 (20%) cases included in the study. These were either hemangioma or vascular malformations.

Majority of the cases (*n* = 31) included in the study showed hypointense signal on T1-weighted images. Only four lesions showed hyperintense signal on T1-weighted images. All these lesions show hypointensity on fat suppressed images. Out of these, 3 were lipoma of the thenar and hypothenar eminence; the fourth case was a cavernous haemangioma with predominant fatty component. T2-weighted hyperintensity was noted in 85% (*n* = 30) of the lesions. Three lesions were hypointense on T2-weighted images. All these lesions were diagnosed as GCTTS on histopathology. Rest of the lesions were isointense on T2-weighted images.

Only 7 (20%) of the cases were nonenhancing. Postcontrast enhancement was seen in the rest of them. It was further divided into solid, peripheral enhancement and patchy enhancement. Most common pattern was patchy enhancement seen in 16 (45%) cases. Vascular lesions showed intense patchy progressive enhancement. The nonenhancing lesions consisted of lipomas (*n* = 3), ganglion (*n* = 3), and benign fibrous histiocytoma (*n* = 1). Glomus tumors and GCTTS showed solid enhancement. Peripheral enhancement was seen in chronic tenosynovitis and also in case of tubercular dactylitis with collection.

A correct diagnosis was possible on MRI in 80% of the cases (*n* = 28). The incorrect diagnosis included benign fibrous histiocytoma (*n* = 2), synovial sarcoma (*n* = 2), benign spindle cell tumor (*n* = 2), and hamartomatous lesions (*n* = 1).

### 3.3. Distribution of Cases according to Cytology/Histopathology

A total of 13 histologically different cases were included in the study (Table 1). The maximum number of cases comprised vascular malformations and hemagioma.

## 4. Discussion

Clinical assessment of the palpable lesions of hand is of utmost importance. Imaging is used for confirmation of the clinical diagnosis and delineation of lesion extent. The most common swellings are of ganglion, synovial, and peritendinous origin [5]. The imaging features of the ganglions are typical, therefore making MRI an important tool for diagnosis. One has to look for the presence of soft tissue components and abnormal signal intensity to rule out other diagnoses. Many of the soft tissue masses have nonspecific appearance on imaging. However some of them like lipoma and GCTTS have typical imaging features.

In a study done by Capelastegui et al., MRI and case records of 134 patients presenting with swellings in the hand and wrist were reviewed [7]. In 126 cases, cause of the swelling could be demonstrated in the study. In 8 patients, no focal lesion was seen. In their study, ganglions were the most common cause of the swelling accounting for 36 (26.86%) cases. Vascular lesions were seen in 9 cases and were the most frequently encountered lesions among soft tissue tumors [7]. However in our study, the most common cases presenting with hand swellings were hemangiomas/vascular malformations.

Hemangiomas on USG show iso/hyperechoic well-defined lesions with anechoic areas in between with/without color flow [8]. The hyperechoic areas likely represent fatty component. Hemangiomas that usually have a well-defined margin but may show infiltrative margins [9]. They typically have intermediate signal intensity on T1-weighted images and high signal intensity on T2-weighted images [9, 10]. However, intralesional heterogeneity can be produced by reactive fatty tissue around the neoplastic vessels (hyperintense on T1W images), by blood vessels (hyperintense on T1W images) or by intralesional haemorrhage (variable signal intensity depending on the duration). In addition, phleboliths are seen as low signal intensity foci on both T1W and T2W images [10]. There is avid postcontrast enhancement with visualisation of feeding vessels. In a study by Theumann et al., MRI features were reported in 15 patients with hemagiomas of fingers [11]. Typical MRI findings were recorded in 10 patients and atypical findings in 5 patients. Atypical features included mass like appearance with homogeneous diffuse enhancement (*n* = 2) and poor heterogeneous enhancement (*n* = 3). We performed MRI in 4 lesions proven to be hemangiomas [11]. In one of the cases, there was fat component seen as T1W hyperintense component with suppression on fat suppressed images. In all the cases, patchy intense enhancement was seen.

**Figure 1:**
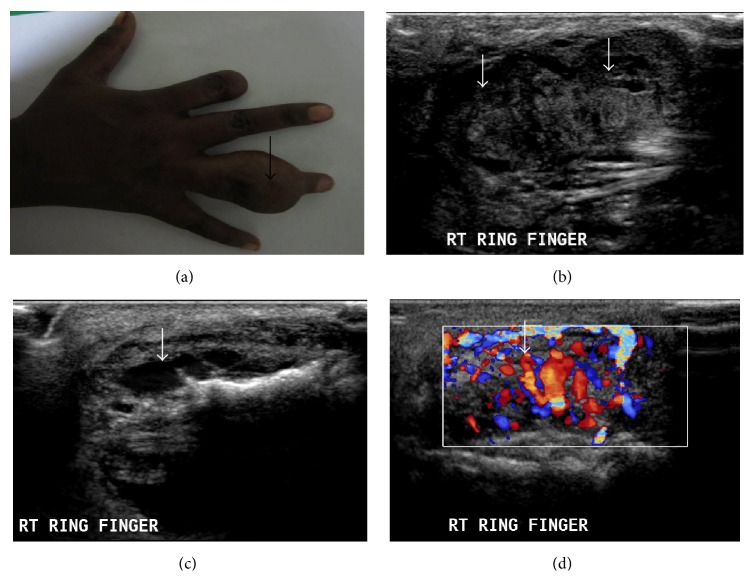
AVM. A 22-year-old female presented with compressible swelling involving middle phalanx of right ring finger ((a), arrow). Ultrasonography shows a heterogeneously hypoechoic lesion with multiple prominent linear hypoechoic channel (arrows) and irregularity of underlying bone ((b), (c)). Doppler showed markedly increased intralesional vascularity ((d), arrow).

**Figure 2:**
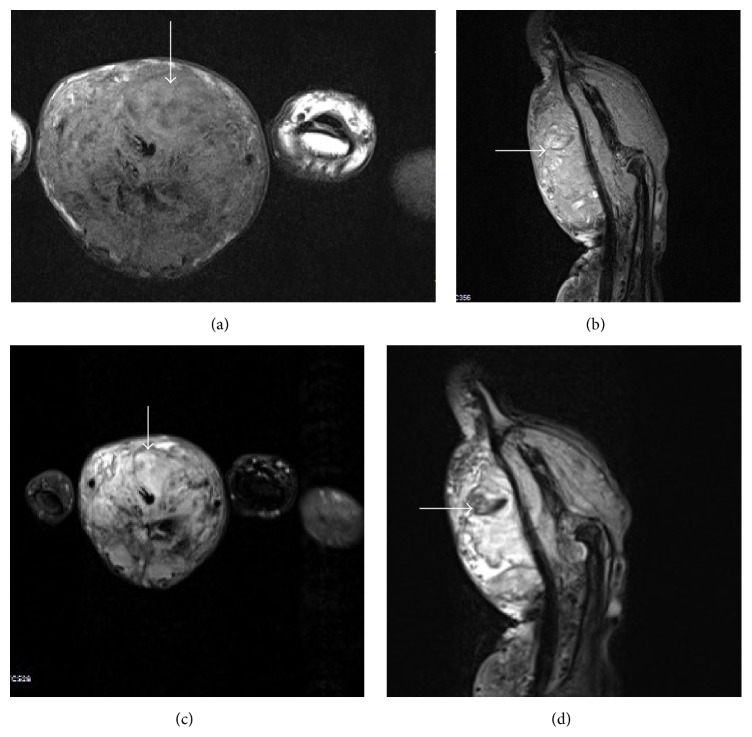
AVM. MRI (of the same patient as in Figure 1) shows a large heterogeneous T1W hypointense (a) and T2WFS hyperintense (b) lesion (arrows) showing few intralesional flow voids, marrow extension, and cortical breech. On post contrast T1WFS sequences ((c), (d)) the lesion shows intense heterogeneous postcontrast enhancement (arrows).

Vascular malformations are classified into high flow and low flow [8, 12]. The latter is further classified into venous, lymphatic, capillary, and venocapillary forms based on the predominant component. Slow flow vascular malformations are usually septated and show intermediate to low signal intensity on T1W and high signal intensity on T2W images. The lymphatic malformation tends to be more infiltrative than the venous form and is more likely to demonstrate fluid-fluid levels [12]. The venous malformation is characterised by phleboliths seen as low signal foci on all pulse sequences. The lymphatic malformation shows no enhancement (microcystic) or septated enhancement (macrocystic). The venous form shows delayed venous enhancement. No arterial feeder (seen as flow voids), draining vein, and arterial or early venous enhancement are seen in low flow vascular malformation. The latter are the characteristics features of the high flow AVM [12]. The lesions can show intraosseous extension in either vascular malformation. In our study, we evaluated 5 vascular malformations in the hand. Flow voids were seen in three cases. All these cases also showed arterial feeders suggesting a diagnosis of high flow vascular malformation (Figures 1 and 2). Underlying bone changes were present in two cases. Two cases showed phleboliths. In all the cases included in our study the diagnosis of vascular malformation could be made on USG/Doppler alone; however MRI better defined the extent of the lesion, underlying bone changes, and involvement of marrow.

We encountered 3 cases of ganglions in our study. Imaging is important in some cases to look for atypical findings, solid components, heterogeneous contents, and abnormal SI, in the surrounding tissues [5]. De Flaviis et al. conducted a study on 14 patients presenting with soft tissue swellings of the hand [13]. Clinical diagnosis was kept as ganglion in 13 cases. High resolution USG was done in all the cases. Ganglions appeared as well-defined anechoic lesions with thin wall in recent cases. In older lesions, internal echoes and thicker walls were identified. Presence of a liquid filled duct directed towards articular surface is considered diagnostic [14]. A typical lesion on MRI shows fluid signal intensity with subtle peripheral enhancement. Atypical findings presenting a diagnostic challenge include debris, haemorrhage, synovial thickening, or cyst rupture producing diverse MRI appearances [15]. In our study, no atypical findings were noted.

**Figure 3:**
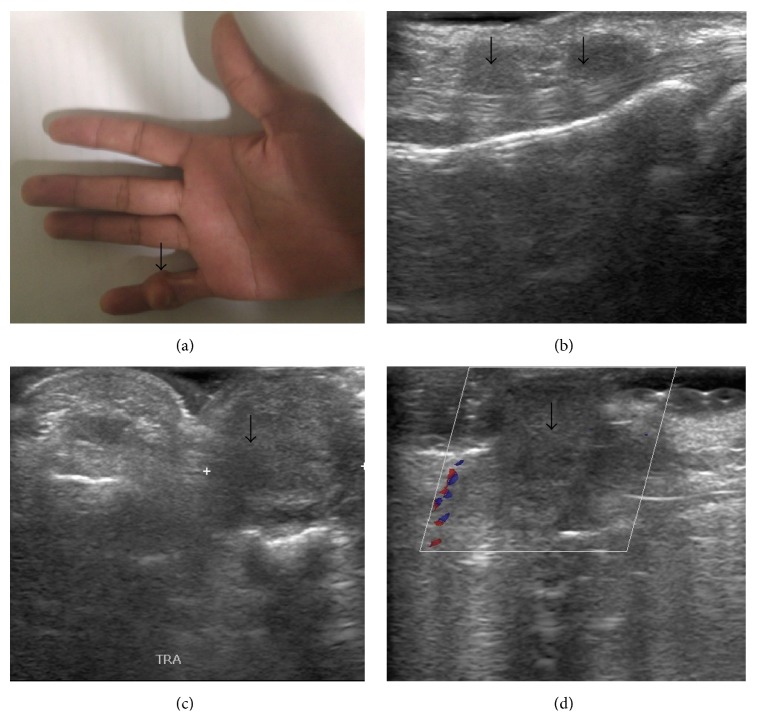
Giant cell tumor of tendon sheath. A 20-year-old female presented with swelling in right 5th finger over middle phalanx ((a), arrow). Ultrasonography and Doppler show multiple well-defined, hypoechoic, hypovascular lesions along the flexor tendons ((b)–(d), arrows).

**Figure 4:**
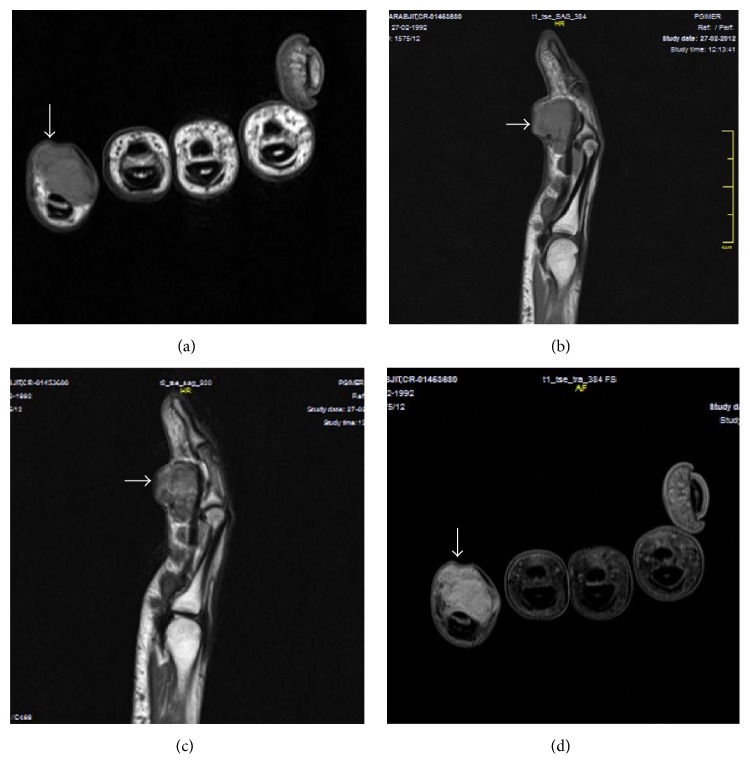
Giant cell tumor of tendon sheath. MRI (of the same patient as in Figure 3) show multiple, well-defined, lobulated, T1W, and T2W hypointense lesions ((a)–(c)) along the flexor tendons of right 5th finger. These lesions show mild enhancement seen on postgadolinium T1FS images (d).

GCCTS are the second most common lesions of the hand and wrist. These present as slow growing, firm, nontender mass with a predilection for the radial three digits especially around the distal interphalangeal joint [16]. In a study by Capelastegui et al., GCTTS was the most frequent specific diagnosis [7]. These show characteristic imaging features, low signal on both T1 and T2 WI (due to fibrosis and hemosiderin deposition) with postcontrast enhancement. In 1 case it was located along the carpus [17]. In our study, 3 such cases were diagnosed. On gray scale imaging these were predominantly isoechoic with presence of heterogeneity in larger lesions. No increased vascularity was demonstrated on color flow imaging. MRI showed characteristic findings in relation to the flexor tendon (Figures 3 and 4).

Lipoma has characteristic signal intensity on MRI. It parallels that of subcutaneous fat on all pulse sequences [18]. On gray scale USG they are characteristically hyperechoic with no evidence of vascularity on color flow imaging. In MRI, the lesions show increased signal on all sequences with suppression on fat saturated images.

**Figure 5:**
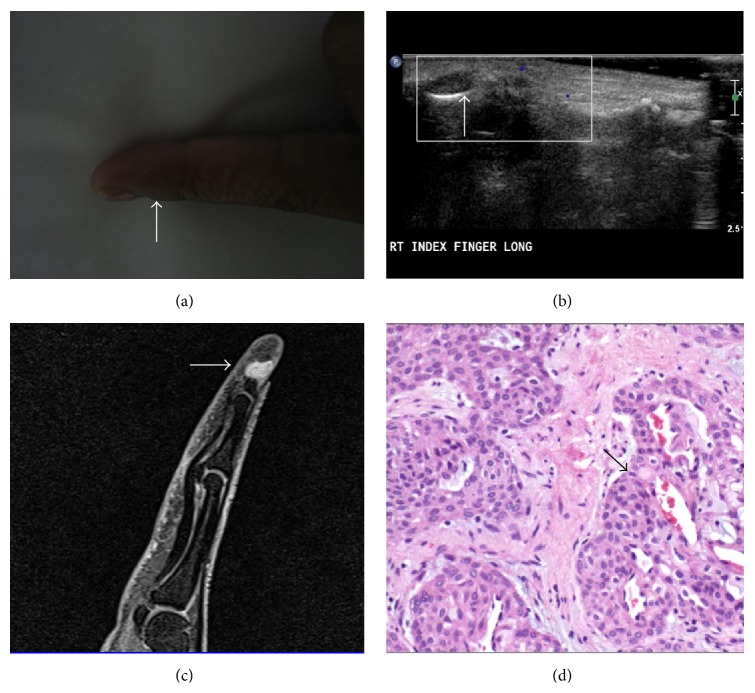
Glomus tumour. A 40-year-old female patient presented with painful, tender, and noncompressible swelling involving the region of distal phalanx of right index finger ((a), arrow). Ultrasonography (b) showed a well-defined, subcentimetric, hypoechoic, hypovascular soft tissue lesion (arrow). Postcontrast MRI (c) shows a small enhancing lesion along the flexor tendon sheath (arrow). Microphotograph (d) shows vascular lumina surrounded by a solid proliferation of glomus cells with perfectly round nuclei and acidophilic cytoplasm (arrow). H&E ×200.

In a study by Montandon et al., imaging features in 8 cases of subungual glomus tumors were evaluated. Doppler ultrasonography was positive in five cases showing a hypoechoic nodule with internal vascularity [19]. Magnetic resonance imaging was positive in all of the cases. It showed solid nodule, hypointense on T1- and hyperintense on T2-weighted image, with homogeneous contrast enhancement [19]. In a study by Trehan et al., MRI findings in 36 of the 46 patients who had pathologically confirmed hand glomus tumor were analysed. A preoperative diagnosis could be established in 24 cases; 7 cases were indeterminate and 5 were negative [20]. Authors concluded that failure to diagnose glomus tumors on MRI could be attributed to atypical pathology, atypical location (i.e., not located in the subungual region), absence of bone erosion, and lack of clinical suspicion [20]. We evaluated 3 glomus tumors in our study (Figure 5). Atypical findings were recorded in none of the patients.

**Figure 6:**
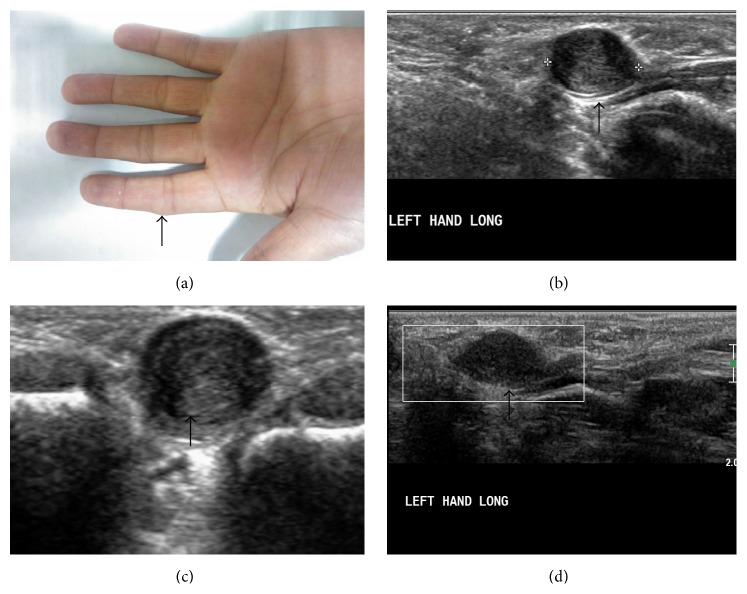
Schwannoma. A 25-year-old female patient presented with firm, noncompressible swelling over the radial aspect of the proximal interphalangeal joint of 2nd digit ((a), arrow). Ultrasonography ((b)–(d)) showed a well-defined, oval, hypoechoic, hypovascular lesion (arrow).

**Figure 7:**
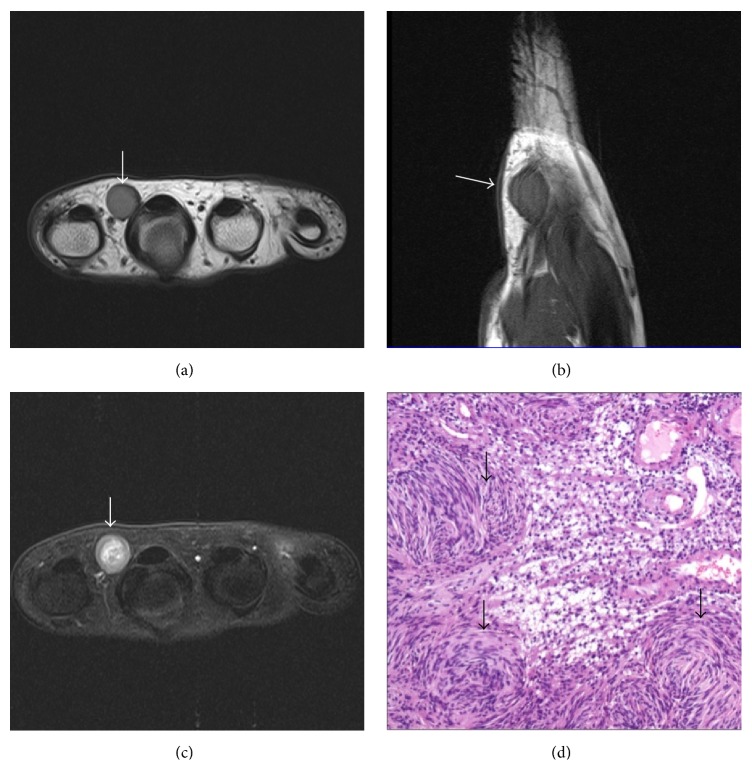
Schwannoma. MRI (of the same patient as in Figure 6) shows a well-defined T1W ((a), (b)) hypointense and T2WFS (c) hyperintense lesion in 2nd-3rd web space (arrows). Underlying bone shows normal cortical outline. Microphotograph (d) shows hypocellular and hypercellular areas with vessels showing perivascular hyalinization (arrows). H&E ×100.

**Figure 8:**
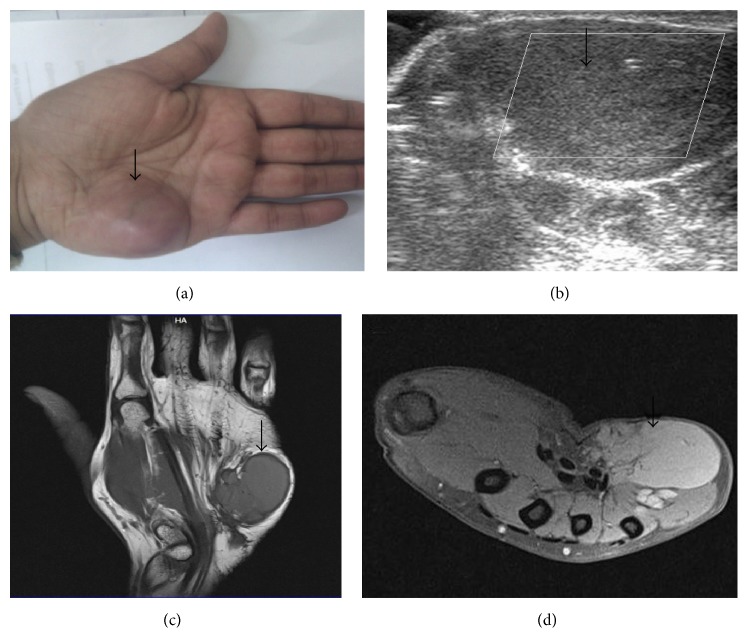
Synovial sarcoma. A 34-year-old female patient presented with noncompressible swelling involving hypothenar eminence of left hand ((a), arrow). Ultrasonography shows a hypoechoic lesion with absent color flow (b). MRI shows a large, lobulated T1W hypointense ((c), arrow) and T1WFS hyperintense ((d), arrow) lesion.

**Figure 9:**
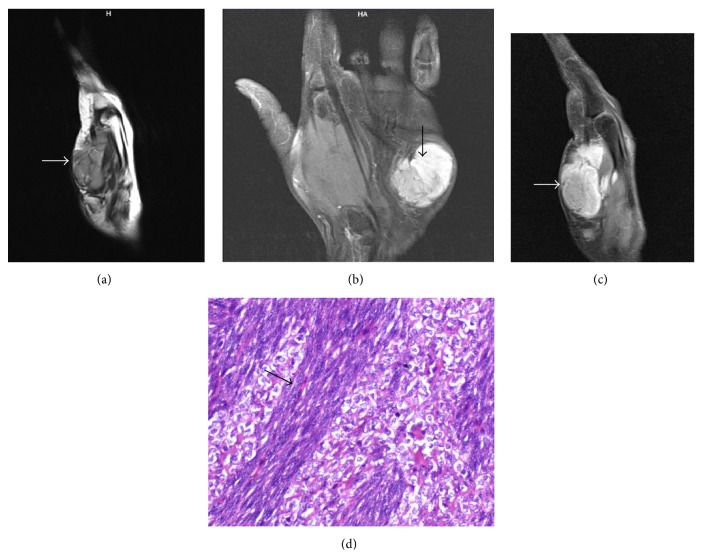
Synovial sarcoma. MRI (of the same patient as Figure 8). On T2W images ((a), (b)) the lesion is hyperintense (arrow). On postcontrast T1WFS images (c) the lesion shows heterogeneous postcontrast enhancement (arrow). Microphotograph (d) shows spindle shaped cells in fascicles (arrow) with intervening plump epithelial cells. H&E ×200.

Neurogenic tumors (Figures 6 and 7), benign fibrous histiocytoma, hamartoma, synovial sarcoma (Figures 8 and 9), chronic nodular tenosynovitis, and tubercular dactylitis with abscess are rare lesions of the hand and their imaging characteristics are similar to those occurring elsewhere in the body [5, 21–25]. Diagnosis of neurogenic tumor on imaging is considered if relationship with a neurovascular bundle can be demonstrated. Otherwise, the imaging can be nonspecific, however, important for delineating lesion extent. Similarly, the other lesions tend to have nonspecific imaging findings. The last two entities, however, have some features that can suggest the possible diagnosis. In nodular tenosynovitis, there is hypointense signal on T1W and T2W images. The bone lesion is the key to the diagnosis of the tubercular nature of the abscess in dactylitis.

## 5. Limitations of the Study

Our study population is small. There is diverse range of pathologies included in our study with insufficient number of cases of certain pathological entities in the included study population, for example, benign fibrous histiocytoma. Pathological confirmation was achieved by FNAC and not by histopathology in some cases.

## 6. Conclusion

Radiological investigations (USG and MRI) are important tools for the diagnosis of the soft tissue swellings of the hand. Relevant clinical history and examination play a pivotal role. Ultrasound is a widely available modality and can be diagnostic in certain entities like vascular malformations and ganglions. MRI can provide conclusive imaging findings in many pathological lesions (vascular lesions, lipomas, GCTTS, etc.) and is important for providing accurate anatomical extent. In certain lesions like glomus tumors, there is limited role of USG and patient can be directly subjected to MRI for the confirmation of the clinical diagnosis. However, pathologic confirmation of the diagnosis is important in some entities especially in absence of characteristic imaging findings.

## Figures and Tables

**Table 1:** Distribution of different types of tumors on HPE/FNAC.

Sr. number	Pathological diagnosis	Number of patients
1	Vascular malformation	5 (15%)
2	Haemangioma	4 (11.5%)
3	GCTTS	3 (8.5%)
4	Ganglion	3 (8.5%)
5	Glomus tumor	3 (8.5%)
6	Lipoma	3 (8.5%)
7	Schwannoma	2 (5.5%)
8	Hamartoma	2 (5.5%)
9	Neurofibroma	4 (11%)
10	Benign fibrous histiocytoma	2 (5.5%)
11	Synovial sarcoma	1 (2.7%)
12	TB	2 (5.5%)
13	Nonspecific inflammation	1 (2.7%)
